# Are Chinese Netizens Willing to Speak Out? The Spiral of Silence in Public Reactions to Controversial Food Safety Issues on Social Media

**DOI:** 10.3390/ijerph182413114

**Published:** 2021-12-12

**Authors:** Linjia Xu, Jiaying Liu, Jarim Kim, Myoung-Gi Chon

**Affiliations:** 1 School of Chinese Language and Literature, University of International Business and Economics, Beijing 100029, China; xulinjia@uibe.edu.cn; 2 Department of Communication Studies, University of Georgia, Athens, GA 30602, USA; 3 Department of Communication, Yonsei University, Seoul 03722, Korea; jarimkim@yonsei.ac.kr; 4 School of Communication and Journalism, Auburn University, Auburn, AL 36849, USA; mzc0113@auburn.edu

**Keywords:** genetically modified food, food additives, spiral of silence, public opinion

## Abstract

This study examines the influential factors posited by the Spiral of Silence Theory (SoS) in shaping people’s perceptions of the overall public opinion towards food safety issues in China and their willingness to speak out. Two highly controversial issues, including genetically modified (GM) food and food additives, are examined. Using an online opt-in panel in China, we collected survey responses from a total of 1089 respondents, with a comparable age distribution to that of Chinese netizens, as indicated in the most recent census. Ordinary Least Squares (OLS) regressions were conducted to make statistical inferences about the proposed research questions and hypotheses. Findings suggest that perceived opinion incongruence, self-relevance, and self-influence significantly affected the extent to which people were willing to express their opinions on social media for the genetically modified food issue, but not the use of food additive issue. The study provides evidence of the silencing effect on publicly expressing opinions about the food safety related issues in China and clarifies the potential boundary conditions of the SoS mechanism in the context of Chinese social media where the majority of public opinions come into formation.

## 1. Introduction

Social media has become the important platforms to engage the public in science and health related issues by facilitating online dialogues and imparting scientific knowledge [1,2]. These interactive channels that feature user-generated content may profoundly shape public perceptions towards some vehemently debated issues, such as genetically modified foods (GM foods), use of food additives, or vaccination [3,4]. Despite scientists’ best efforts to communicate science, health, and risk information accurately, public misperceptions persist [5]. The very first step to address such misperceptions is to know what the public may have misunderstandings about. If people are willing to speak out and engage in science discussions on social media, we would be able to know what their perceptions are, whether those perceptions are accurate; and, if no, the prevalence of the misperceptions held in the public and where our priority lies. Thus, we consider promoting public conversations and engagement in the online sphere as a necessary initial step to ultimately help achieve our goals of effectively improving public understanding of science and promote effective public deliberation.

In recent years, food related issues have received increasing media coverage and scholarly attention in China [6]. New patterns of public engagement, such as active debates and deliberation about these issues, have emerged, facilitated by the rapidly increased use of the Internet and proliferation of social media and apps in China [7]. Informed by the Spiral of Silence Theory [8], the current study tries to examine whether Chinese people would openly express opinions about controversial food issues online, and what factors may contribute to their decision to speak out. Specifically, we investigate this question in the topical contexts of GM foods and food additives, which are currently considered as the most salient and controversial food safety issues in China [6].

### 1.1. Genetically Modified Food and Food Additive Issues in China

Food safety is everybody’s concern. In China, genetically modified (GM) food safety and the use of food additives are ranked at the top of the list that has elicited most public debates and anxiety, reflecting people’s concerns about the role of technology in food production and its subsequent long-term consequences [9].

The dispute on GM technology is a worldwide phenomenon, despite that scientific findings converge to suggest that the risks of commercial GM foods are not larger than their traditional counterparts [10]. In China, the government approved commercial planting of GM cotton in 1997. Since then, GM technology has been gradually gaining the public’s attention. New technologies are known to evoke public anxiety regarding how they may affect the status quo by reshuffling the distribution of social roles and responsibilities, challenging human capacities, moral norms, and valued identities [11]. For GM technology, in particular, people also worry about it being “unnatural” or tampering with the natural law that governs all living organisms [12]. Its long-term impacts on human health, living environment and the ecological system are also deemed uncertain [13,14]. In 2013, a well-known public debate on GM foods between two celebrities, TV host Yongyuan Cui and science writer Zhouzi Fang, attracted massive media coverage and public discussions. Researchers observed that netizens, i.e., those who exhibited habitual and avid use of internet, started to hold predominantly negative opinions and perceptions toward GM foods and reacted to the issue in an increasingly emotional way after the debate, while, paradoxically, knowing very little about it [15]. The percentage of Chinese citizens who considered GM foods to be safe dramatically decreased from 35% to 11.9% from 2002 to 2016 [16,17]. Most Chinese citizens currently hold skeptical attitudes towards GM foods, and such perception was not formed through a thorough understanding of risks and benefits associated with GM foods; instead, it was primarily shaped by the celebrity debates and media reports that prevailed in the public communication environment [7].

In China, doubts about the use of food additives concern illegal usage, overuse, and use of banned additives. Issues, such as melamine in dairy products, the use of carcinogenic dye “Sudan Red”, and clenbuterol in pork, attract public attention nationwide. These landmark events provoked high anxiety among the public, who have limited knowledge to distinguish between legal and illegal food additives [18,19]. Furthermore, the illegal use of food additives has become the primary cause for warnings against Chinese foods exported to the United States, Japan, and Korea [20]. While Chinese consumers were aware of the benefits some food additives may offer [21], in general, food additives were considered unnatural, artificial, containing harmful chemicals, and potentially posing health risks to human health [22], especially among those with lower education levels [6]. In 2017, Chinese government urged to promote scientific understanding of food additives and requested the food industry to strictly abide by relevant regulations [23]. As a result of this concentrated effort, many Chinese started to show more support of the appropriate use of legal food additives [19].

### 1.2. The Spiral of Silence in the Social Media Age

The Spiral of Silence Theory (SoS) sheds important light on public engagement and opinion formation processes [8]. It posits that individuals determine whether to voice out their own opinions by gauging the surrounding opinion climate. The theory has been examined in a variety of topical contexts, including political opinions and election outcomes [24,25], same-sex marriage [26], abortion [27], childhood vaccination [28], and GM foods [4]. These studies were generally supportive of the theory, though the effect sizes varied [29,30]. Our study extends this line of inquiry and examines public expressions in scientific issues in China.

While the propositions of the theory have been mainly studied in face-to-face settings or within mass media contexts [31,32], emerging research started to document the existence and scope of the silencing effect in online communication contexts, such as social media [33,34]. Previous research has pointed out that, in both in-person and online settings, people are more willing to share their views if they perceive that their audience agrees with them [35]. Important questions related to how individuals engage in public discussions about controversial issues online still await to be examined. For example, will the perceived opinion of netizens induce the silencing effect? Or will the online environment, which often facilitates anonymity, help diminish social pressure and, in turn, promote individuals’ outspokenness?

### 1.3. Perceived Opinion of Netizens and Self-Opinion on Perceived Opinion of General Population

The key premise of the SoS theory is that perceived opinion of general population may affect people’s willingness to express their own opinions. Four types of opinion perceptions are often considered as exerting powerful influence on individuals’ decision making in their daily life and on their opinion expression online: current, future, important others’, and experts’ opinions. Present opinion refers to individuals’ perceptions of the current majority opinion of the general public [8], including not only the individual netizens but also various organizations or entities with powerful presence and influence on the internet. Likewise, future opinion refers to individuals’ perceptions of the majority opinion of the general public in the near future. In addition, research also suggested the importance of the perceived opinion of important others, such as families and friends, which may outweigh that of the anonymous general public [32,36]. According to the China Internet Network Information Center, 97% of Chinese netizens are currently using social media, with the primary motivation to be staying connected with families and friends [37]. The most commonly used social media (by 87% of netizens) is WeChat, a “super-app” that allows for instant messaging and information sharing with families and friends, as well as provides all types of news and information, including science and health related topics [37]. In the context of China, thus, we consider the perceived opinion of important others to play an important role in the silencing effect process. Finally, authoritative or professional opinions from experts and opinion leaders often dominate in online discussions, and such social status cues may negatively influence netizens’ willingness to speak out [38]. We, thus, also examine the perceived opinion of experts to more comprehensively understand how these factors may influence netizens’ willingness to speak out on social media.

It is less known, however, what factors may contribute to the formation of such opinion perceptions in the age of social media. Given the increasingly central role that social media and the Internet, in general, plays in informing the public about what happens in the world, particularly in the topical areas that are controversial and scientifically uncertain, we speculate that people’s perceptions about what the majority of netizens believe, i.e., perceived opinion of netizens, may strongly shape their assessment of the overall opinion about food safety issues in China. This question is of particular importance to be examined in the Chinese context, given the particular realities that currently exist on the Chinese Internet, such as strict censorship and the fact that some online opinion leaders are paid to lead opinions in certain ways. The following hypotheses are put forth:

**Hypothesis** **1.** *Perceived opinion of netizens is positively associated with (a) perceived present opinion in the general public, (b) perceived future opinion in the general public, (c) perceived opinion of important others, and (d) perceived opinion of experts about food safety issues on social media.*

People are also more likely to publicly express their thoughts when they hold strong to their own opinions, i.e., self-opinions. Kim and colleagues found that personal opinion was positively associated with perceived support for GM foods in Korea but did not directly tap into how it may shape people’s estimates of public opinions and their subsequent communicative outcomes [4]. We propose to further examine the effect of one’s self-opinions to help advance understanding toward how self-opinions may affect the assessment of opinion climates, as well as to establish the absolute effect of this potentially important factor on communicative actions online.

**Hypothesis** **2.** *Individuals’ self-opinion is positively associated with (a) perceived present opinion in the general public, (b) perceived future opinion in the general public, (c) perceived opinion of important others, and (d) perceived opinion of experts about food safety issues on social media.*

### 1.4. Factors That Influence Willingness to Speak Out

Perceived Opinion Congruence. A key proposition in the SoS theory is the relationship between perceived opinion (in)congruence and willingness to speak out. If one perceives that their own opinion contradicts or is incongruent with the opinion climate, it is likely that one’s willingness to express own opinions in the public will be inhibited [8]. Previous empirical research examining this proposition has revealed inconsistent findings. While some research found significant and positive associations between opinion congruence perception and willingness to speak out [4,32,39,40], others observed either very small effect sizes (e.g., average r = 0.054, 95% CI [0.028, 0.080] in [39]) or non-significant results [26,41]. This theoretical proposition warrants further examination in our study context. As discussed earlier, given the existence of different types of opinion perceptions, we plan to evaluate perceived opinion congruence comprehensively by taking into consideration all the different types of opinion perceptions.

**Hypothesis** **3.** *Opinion incongruence between self-opinion and opinion perceptions is negatively associated with willingness to speak out about food safety issues on social media.*

Self-Relevance and Self-Influence. Issue relevance to oneself was often found to be a strong predictor of public opinion expression [4,42]. Individuals may be more active in communicative actions, either to endorse, defend, or argue against online others when they feel strongly about food safety related issues. Considering that heightened self-relevance may motivate individuals to obtain more information and pay more attention to food safety related facts, knowledge, discussions, and opinions from different sources and may, thus, influence their public opinion estimation, it is also interesting to explore whether self-relevance would affect opinion perceptions and to what extent. We, thus, propose the following research question and hypothesis,

**RQ1:**  What is the relationship between self-relevance and (a) perceived present opinion in the general public, (b) perceived future opinion in the general public, and (c) perceived opinion of important others about food safety issues on social media?

**Hypothesis** **4.** *Self-relevance is positively associated with willingness to speak out about food safety issues on social media.*

In addition, the modern view of online social influence de-emphasizes the role of influencers or opinion leaders defined by conventional standards [43]. It posits that any online users can be influencers, especially those that are connectors (people who are well-connected with social others), mavens (people who offer new information or perspectives), or salespeople (people who can persuade) [44,45]. Users with these traits are more likely to disseminate information, engage in discussions, and express opinions on social media [43,46]. We, thus, hypothesize that individual differences in people’s capability of influencing others can be an important factor contributing to their decision to speak out about food safety issues online:

**Hypothesis** **5.** *Self-influence is positively associated with willingness to speak out about food safety issues on social media.*

## 2. Materials and Methods

### 2.1. Sample and Procedures

An online survey was conducted in January–February 2018 through SoJump online survey company. SoJump is one of the biggest online survey companies in China with its nationwide sample pool, and operates similarly to Amazon’s Mechanical Turk [47]. To ensure data quality, the study used Sojump’s paid sample service, which possesses a panel of more than 2.6 million respondents from different cities in China. The profile of the SoJump panelists is representative of Chinese netizens and reflects their patterns in social media use [48]. The respondents get immediate reimbursement (points redeemable for cash) once they complete the study. To ensure answer quality for this study, SoJump added in foil questions and manually checked a random sample of answers from the participants. This study has passed the research ethics review in the authors’ institution.

The final sample size used for analysis is 1089. The participants were from a wide range of age groups, whose distribution is comparable to that of the Chinese netizen sample reported in the most recent censuses conducted in China [49], suggesting that our sample is more indicative of opinions and ideas of Chinese netizens, rather than the general population. On average, the respondents spent 5.14 h per day on the Internet. Furthermore, 97% of them obtained information related to GM foods and food additives from social media. Respondents also reported their most preferred media channels (including TV news, website, news apps, social media, newspaper, radio, magazine, NGO brochures, government brochures) on a 5-point scale (1 = strongly unfavorable, 5 = strongly favorable). Social media was among the top three choices for obtaining both the GM foods (M = 3.62, SD = 1.02) and the food additives information (M = 3.60, SD = 1.02).

### 2.2. Measures

Perceived opinion of netizens. Perceived opinion of netizens was measured with two questions asking about people’s perceptions of how netizens and the internet discussions in general, feel about the two food safety issues on a 5-point scale (1 = strongly unfavorable, 5 = strongly favorable). The two items were then combined into a single variable for analysis.

Perceived opinion of general population. We adapted measures from Kim et al. [4] to assess respondents’ perceived present and future opinions of the general population, as well as perceived opinions of important others and experts. Perceived present opinion among the Chinese general population was measured by asking respondents their perceptions of Chinese people’s present opinion on the food safety issues. Perceived future opinion was measured by asking respondents to gauge the public opinion toward the food safety issue in the next 10 years. Perceived opinion of important others was measured by asking the participants to gauge the opinions of their friends and family members on the two issues. Perceived opinion of experts was measured by asking the participants to gauge the opinions of experts (e.g., from government agencies, universities, and research institutions). All perceived opinion of general population variables were measured on a 5-point scale (1 = strongly unfavorable, 5 = strongly favorable).

Self-opinion. Participants’ own opinions about the food safety issues were adapted from previous GM opinion research [14] and measured with five items (e.g., “How do you feel about GM foods/food additives in general?” “I think GM foods are ethically problematic”) on a 5-point scale (1 = strongly unfavorable, 5 = strongly favorable). Of note, the ethical concerns associated with GM technology discussed in the Chinese context include potential unintentional or adverse effects of the generation of new species on untargeted neutral or beneficial species, and long-term effects on human health, ecosystem, and ownership of food supply [50]. These items were averaged into a single variable for analysis.

Self-relevance. We measured self-relevance with two statements (e.g., “I see a close connection between me and this issue.”) on a 5-point scale (1 = strongly disagree, 5 = strongly agree). The two items were then averaged to create a single variable for analysis.

Self-influence. We adapted measures from Boster’s scale [44] to assess respondents’ self-influence capabilities. Respondents were asked to rate their level of agreement (1 = strongly disagree, 5 = strongly agree) on five statements (e.g., “I can lead others to accept my point of view if I want.”). These items were then combined into a single variable for analysis.

Willingness to speak out. The focal dependent variable, individuals’ willingness to speak out on a social networking site, was measured with three items adapted from Stoycheff’s study [51]. Participants were asked to indicate on a 3-point scale (1 = no, 2 = unsure, 3 = yes) whether they would (a) compose posts, (b) repost or retweet posts, and (c) comment on posts related to the two food safety related issues. We then averaged the items to produce the willingness to speak out variable for the two issues for analysis.

We also measured demographic variables, including age, gender, education, and monthly household income (see Table 1 for details). These variables served as control variables in the regression models.

## 3. Results

Table 1 shows the sample demographic distributions.

Our first hypothesis (H1) examines whether and to what extent the Internet plays a role as a source of information that people use to assess the opinion climate toward the two food safety issues amongst the general Chinese population, and people who are close to them. In addition, we are also interested in understanding how individual difference factors, such as self-opinion and self-relevance, may contribute to the formation of opinion perceptions (H2 and RQ1). To answer these questions, four measures of perceived opinion of general population for the GM food issue and the food additives issue (present and future opinions among general population in China, as well as opinions among important others and experts) were examined as dependent variables. To take into consideration of different aspects and sources of opinion perceptions, we first calculated the absolute differences between self-opinion and different types of opinion perception variables, including perceived present opinion (GM foods: M = 2.43, SD = 0.67; food additives: M = 0.81, SD = 0.62), perceived future opinion (GM foods: M = 2.04, SD = 0.78; food additives: M = 0.82, SD = 0.64), perceived opinion of netizens (GM foods: M = 2.38, SD = 0.70; food additives: M = 0.78, SD = 0.60), perceived opinion of important others (GM foods: M = 2.48, SD = 0.64; food additives: M = 0.90, SD = 0.65), and perceived opinion of experts (GM foods: M = 3.07, SD = 0.68; food additives: M = 0.94, SD = 0.71). These absolute difference variables were then averaged and combined into a single opinion incongruence variable for analysis (GM foods: M = 2.48, SD = 0.76; food additives: M = 0.85, SD = 0.53). Table 2 lists detailed descriptions and statistics for the above focal variables introduced.

We then ran OLS regression models with perceptions of netizens’ opinion, self-opinion, and self-relevance serving as independent variables, and demographics as control variables (Table 3).

As summarized in Table 3, perceived opinion of netizens was significantly associated with perceived present opinion, future opinion among general Chinese population, perceived opinions among important others, and perceived opinion among experts. H1 was supported, suggesting the role of perceived opinion of netizens as an important source of information for people to form an overall impression on public opinion. Self-opinion was also found to be positively associated with all perceived opinion of general population variables for both issues. H2 was supported. For RQ1, we observed that self-relevance was negatively associated with perceived present opinion of the general public and opinion of important others, which may indicate that the more relevant the food safety issues are to oneself, the more likely they will encode, memorize, and react more strongly to negative information about the issues, which in turn shape their perceptions of social reality. Interestingly, self-relevance was found to be positively associated with perceived experts’ opinions on the GM food issue, which may indicate that the more relevant one finds GM food issue to him/herself, the more likely they will perceive that the experts would hold a positive opinion about GM foods. This finding may reflect people’s conformation bias that propels them to interpret experts’ opinion in a positive light, given the importance and relevance of the issue in their daily life. We also observed significant associations between age and perceived present opinion, future opinion, and opinion of experts. Specifically, compared to younger generations, older people perceived more negative present opinion toward the two food related issues, and they were also more pessimistic about future opinion trends and experts’ opinion.

Our next set of hypotheses examined whether perceived opinion congruence, self-relevance, and self-influence, would affect one’s willingness to express their opinions on social media. We fitted an OLS regression model with willingness to speak out as the dependent variable, and the aforementioned factors as independent variables, controlling for demographics (Table 4). As can be seen from Table 4, for the GM food issue, all hypothesized focal independent variables were significantly associated with the dependent variable. Specifically, perceived opinion incongruence was negatively associated with willingness to speak out (β = −0.07, *p* < 0.05; H3), suggesting that more perceived incongruence between one’s own opinion and the perceived opinion of general population toward GM foods, the less likely that they will be willing to voice their opinions about this issue on social media. Self-relevance (H4) and self-influence (H5) were also observed to be significantly and positively associated with willingness to speak out as hypothesized. For the food additive issue, although the association between perceived opinion incongruence and willingness to speak out was also negative, such association, was not significant; H3 was partially supported. Interestingly, self-relevance was found to be negatively associated with willingness to speak out about food additives; H4 was partially supported. Self-influence was significantly and positively associated with willingness to speak out, supporting H5. We provide further discussions regarding these results in the next section.

## 4. Discussion

### 4.1. Theoretical Implications

First and foremost, our study is among one of the pioneering efforts to provide evidence of the silencing effect on publicly expressing opinions about the food safety related issues in the context of Chinese social media platforms, where the majority of public opinions in China comes into formation. We observed that people’s perceived opinions expressed in the Internet environment by the Chinese netizens are closely connected to their view of the overall opinion climates. Despite the unique characteristics of the Internet environment, such as anonymity and heightened user agency, our results indicated that it might not have helped effectively diminish the social pressure that keeps citizens from expressing a minority view. These results are consistent with another study conducted in the context of an Asian country, which confirmed the critical role of Internet in shaping public opinion about the GM food issue, and its silencing effect on public expression in Korea, as well [4]. Together, these findings lend strong support to the predictions from the Spiral of Silence theory in our current new media landscape amongst populations that prioritize interpersonal connections and social values. However, the results observed in this study cannot be generalized to the general Chinese public. Examining the spiral of silence effect in a more representative general Chinese population warrants further examination in the future.

Second, an interesting finding this study revealed was that, while the perceived opinion incongruence was significantly and negatively associated with willingness to speak out for the GM food issue, such association was not observed for the food additive issue. Unlike the common expectation that social pressure would exert a strong influence in collective cultures regardless [52], the findings of the current study remind us that it is important to take into account the nature of the target issues under investigation. Close scrutiny of the self-opinions toward the two issues in our sample indicated that, on average, people held a significantly more negative opinion towards the GM food issue (M = 2.60, SD = 0.70) compared to that of the food additive issue (M = 3.18, SD = 0.68; t(1088) = −24.34, *p* < 0.001). Considering that effects of social pressure emanating from others’ opinions or behaviors are most pronounced under conditions of uncertainty and ambiguity [53], it is possible that the influence of perceived discrepancy in self- and public-opinions is most likely to be observed in situations where the target issue under investigation (i.e., GM foods) possesses these characteristics. Food additive issue, on the other hand may be perceived as more neutral and benign, and less controversial in nature; thus, observed opinion difference may not contribute to people’s decision to express themselves so strongly. Given that most previous studies examining this association were only able to examine one issue or one behavior domain, the two issues examined in this study and the interesting distinctive patterns we detected substantially advanced our understanding of this focal hypothesis in the spiral of silence theory by illuminating a potential boundary condition of how perceived opinion congruence exerts influence, the perceived uncertainty associated with the target issue.

The reasons why Chinese netizens still hold relatively negative opinions towards GM foods, compared to that of food additives, may be attributed to media, institutional and cultural factors. Empirical research examining Chinese media coverage about food safety related issues revealed that almost all major mainstream and new media channels tended to depict GM foods in a negative light and with a cautious tone, whereas reports about food additives included comparatively more rational and objective analyses by distinguishing food additives with illegal and legal ones, and pointed out the acceptability of the latter [54]. In addition, given that the majority of Chinese netizens lack of accurate GM knowledge and tend to politicalize the GM food issue by associating it with government or institutional endeavors rather than commercial matters [7,55], their decision to support or oppose GM foods often goes hand in hand with their trust in the government’s good intentions and capabilities of properly handling issues that are closely relevant to people’s daily life [56]. On the other hand, the food additive issue was associated more often with decisions from the private sector than government institutions. Cultural factors may also to some degree contribute to people’s vigilance toward the GM foods. Chinese traditional culture prioritizes harmony between human and nature and advocates for minimal human involvement or intervention on how living organisms should grow and evolve in their natural habitat. It is, thus, hard for those who are keen on this belief to accept any “unnatural” modification to the environment, to the ecosystem, and to the food products they consume every day [57]. Food additives are considered relatively less of a direct threat to this traditional cultural ideology compared to the GM foods in this regard. It is worth noting that, however, the perceived netizens’ views toward GM foods were not extremely negative, compared to the public sentiment back in 2013 immediately after the debate. This may have reflected the public knowledge change and gradual acceptance of GM food over the past years [16].

The findings of this study also advance our understanding of the important role of both the sociocultural and individual level factors in predicting one’s likelihood to speak out about scientifically uncertain issues. Previous research has emphasized the profound influence of sociocultural values and norms on people’s public behaviors and communicative actions [58,59]. Given that the Chinese culture prioritizes interpersonal connections and social values, we suspect that the silencing effect can be more pronounced in the Chinese context. In other words, it is possible that the specific sociocultural background under investigation can moderate the silencing effect as proposed in the spiral of silence theory. While the current study cannot directly speak to this possibility given that only one sociocultural context (i.e., China) was examined, it may be a promising future direction to examine and compare the silencing effect in two or more cultures simultaneously. In addition, one’s self-opinion is in general a strong predictor of willingness to speak out in previous studies [4]. Opinion congruence, as a focal construct proposed by the spiral of silence theory, was operationalized in this study as the absolute difference between self-opinion (which reflected individual-level determinant) and perceived opinion of the general population (an indicator of influence coming from the surrounding social and cultural environment), well capturing the interplay between individual and sociocultural factors in determining people’s decision to express their opinions or not. Our findings that opinion incongruence can significantly and negatively affect people’s willingness to speak out, particularly for the GM food issue, corroborated the idea that it is not a single factor alone, but the dynamics between the sociocultural and individual sources of influence that jointly determines Chinese netizens’ willingness to speak out.

Besides, our findings also confirmed that individual differences in people’s ability to influence others were significantly and positively associated with their outspokenness online. This individual trait factor may, thus, serve as an important source (with a substantially larger effect size as can be seen from Table 4) to combat external or environmental influence, such as a perceived incongruent opinion, to promote public engagement.

Last but not the least, the results associated with self-relevance showed different patterns for the two issues. Self-relevance had a positive association with willingness to speak out on the GM food issue but a negative association on the food additives issue. We speculate that the explanation to these findings may still lie in the different nature of the two issues. Given that the GM food issue was perceived predominantly more negatively in China, it was possible that people who identified themselves to have higher self-relevance to this issue may feel a greater sense of legitimacy to voice out concerns in order to change the situation. On the other hand, considering the variety of food additives currently available (including both legal and illegal ones), those who were more self-relevant to this issue (presumably those who were more knowledgeable about the spectrum of different types of additives) may feel more cautious about expressing opinions online that may produce misleading interpretations associated with different additives, which should be analyzed on a case-by-case basis.

### 4.2. Practical Implications

The study findings also offer practical implications for science popularization and health campaign designs, as well as policy-making. The past decade has witnessed a phenomenal growth in the use of Internet, especially social media, in science communication and various health care settings. Encouraging people to voice out their opinions and questions online could help policy-makers and scientists understand what people are thinking about and how the various public opinions regarding scientific topics come into formation. Encouraging netizens to speak out and calling for professionals’ monitoring and engagement to promote the dissemination of scientific educational information are not mutually exclusive; instead, both efforts are necessary for achieving the goal of collaboratively creating a healthy and scientific online public communication environment. On one hand, it is important to be able to hear netizens’ opinions, including questioning voices of science. This is not only important for promoting public awareness, engagement, and enthusiasm in science but also serves as an important venue for professionals to access public understanding of science; for example, what do the public already know? What do they misunderstand? Which misperception is prevalent and needs to be corrected in a timely fashion with more targeted strategies? On the other, given the profound influence of the perceived dominant public opinions online in shaping people’s perceptions and decisions, interventions, such as active participation/discussion and correction of misinformation, are necessary to make sure the public have access to accurate, evidence-based scientific information to guide their decision-making. It is important to note that the professionals will need to be cautious and strategic about not dissuading people from expressing their views online, for example, through honest attempts to draw reasonable conclusions from factual evidence, which can be checked or proved, instead of making value judgments about opinions being right or wrong.

Our study findings highlighted the role of the Internet in shaping people’s perceptions of public opinion, suggesting that science educators, health practitioners, and policy-makers should make productive use of the Internet and social media platforms in the dissemination of scientific and educational information to the public, and in bridging various stakeholders to effectively promote health behaviors and reduce population-level risk. Our results indicated that online information generated by the netizens could strongly shape public opinion perceptions, and willingness to speak out, thus, should be better leveraged to improve public engagement in science. More importantly, our findings indicated that people’s various public opinions are heavily influenced by their perceptions of the netizens’ opinions. In view of this, practitioners in health and science communication should be encouraged to actively monitor and participate in public discussions online to help correct misinformation and collaboratively build healthy and scientific online public communication environment which can subsequently affect public decision-making. Social media platforms, such as WeChat and Weibo, in China can also serve as useful venues for policymakers to identify and encourage public expressions of minority views, thus broadening perspectives of public discourse and promoting efficiency of public deliberation on controversial scientific issues.

Food safety and environmental issues are two of the major scientific issues which have attracted a large-scale public engagement and debates in China [55]. The process of public engagement in science for the former has been mainly achieved through online venues, while the latter has been featured with more offline movements. Considering the heightened significance of food safety issues in China, which have gained increasing attention both nationally and internationally in recent years, it is imperative to further investigate the interplay and transformation between online public opinion formation and offline public actions regarding these issues. This will shed light on strategies and effective efforts aimed at better promoting public engagement in science, provoking public interest in science, and improving science literacy and public understanding in the Chinese population.

### 4.3. Limitations and Future Directions

We would also like to note the limitations of this study. First, due to the novel operationalization of the dependent variable, willingness to speak out on social media, we used a modified scale [51] that included various communicative actions, including composing, reposting, or retweeting and commenting on posts, which yielded a relatively low reliability. Future studies would benefit from further establishing reliable measures of the willingness to speak out construct in the social media context. Second, the measures used in this study assessed broader categories of the GM food and food additive issues. Future research is warranted to examine more refined areas of these issues with specific measures (such as the public opinion and willingness to speak out regarding legal versus illegal food additives). Relatedly, perceived opinion measured used in this study were adapted from prior research [4], with many of these variables being assessed with one measurement item. The adapted instruments were also not validated, either. Future research is recommended to examine these core constructs using scales of high reliability and provide validation evidence for the adapted measures. Third, the sample we recruited in this study was more representative of Chinese netizens, rather than the general Chinese population, limiting the generalizability of our findings. In addition, the digital literacy divides make it more likely that only those more educated individuals tend to opt in online panels and participate in online surveys. Due to the cross-sectional nature of the survey design in this study, only associations, not causations, could be inferred from the findings. Future research would benefit from longitudinal designs or randomized experiments to better establish temporal sequence among variables and causal inferences. It is also highly important, as future next steps, to conduct content analytic work or a qualitative analysis on the opposing voices and specific reasons about trusting/distrusting GM foods, to shed light on the most effective ways in which Chinese netizens’ understanding of science can be improved. Lastly, the influence of perceived opinion of netizens should be contextualized in relation to people’s specific social media diet. Online communities provide various types of interactions and possibilities for one to express their opinions. Different characteristics and affordances associated with different types of social media platforms can have important implications for the potential silencing effect. Our study examined the perceived opinion of netizens in general by asking respondents to consider netizens they may encounter on the Internet as a whole. Future studies are recommended to address the specific types of social media people interact with and examine the silencing effect by different social media platforms.

## 5. Conclusions

Situated in the context of two highly controversial food safety related topics in China, namely GM foods and food additives, this study examines how social media may affect Chinese people’s overall public opinion perception formation and willingness to express their opinions.

To summarize, our results suggested that participants’ perceived opinion of netizens and their own opinions were significantly and positively associated with perceived present and future opinions among the general population, as well as the perceived opinions among important others and experts on both issues, supporting H1 and H2. For RQ1, self-relevance was found to be negatively associated with perceived present opinion of the general public and opinion of important others for both issues, but positively associated with perceived experts’ opinions on the GM food issue. It, however, was not significantly associated with the perceived future opinions of the general public on both issues. For H3 and H4, in the context of the GM food issue, we were able to confirm that, as hypothesized, perceived opinion incongruence was negatively, and self-relevance was positively, associated with willingness to speak out. However, for the food additive issue, although the association between perceived opinion incongruence and willingness to speak out was also negative, such association was not significant; for self-relevance, contrary to our expectation, it was found to be negatively associated with willingness to speak out. Therefore, H3 and H4 were partially supported. Finally, our findings confirmed that people’s perception about their own capability to exert influence on others was significantly and positively associated with their willingness to speak out. Therefore, H5 was supported in both the GM food and food additive contexts.

Through this study, we confirmed the important role of perceived opinion of netizens as a potential source people rely on when assessing and forming overall opinion perceptions about both issues. We observed a significant and negative association between perceived opinion incongruence and individuals’ willingness to speak out about the GM food issue; however, such association was not observed for the food additive issue. Those who were more capable of influencing others’ opinions, in general, were more willing to publicly express their opinions on both issues. Our results also indicated that netizens who reported having higher self-relevance to the GM food issue were more likely to speak out; on the contrary, those who felt more relevant to the food additive issue were less likely to speak out. The study clarifies the potential boundary conditions of the mechanism posited in the spiral of silence theory in the context of Chinese social media, where the majority of public opinions come into formation.

## Figures and Tables

**Table 1:** Sample characteristics (n = 1089).

	N	%		n	%
Gender			Age		
Male	543	49.9	18–29 years	395	36.3
Female	546	50.1	30–39 years	470	43.2
Education			40–49 years	155	14.2
Completed graduate degree	86	7.9	50–59 years	56	5.1
Attending/dropping out graduate program	13	1.2	60 years +	13	1.2
Completed college	853	78.3	Monthly household income		
Attending/dropping out college	64	5.9	<$1225	121	11.1
Completed high school	59	5.4	$1225–2450	559	51.3
Attending/dropping out high school	7	0.6	$2450–6127	366	33.6
Completed middle school	5	0.5	>$6127	43	3.9
Completed elementary school	2	0.2			

**Table 2:** Measurement descriptions and statistics of focal variables.

Variables	Description	Values	M	SD	Scale/Index	
Perceived opinion of netizens						
GM foods	In your view, how do netizens feel about GM foods?	1 = Strongly unfavorable, 5 = Strongly favorable	2.49	0.93	M = 2.64 SD = 0.80 α = 0.65	
	In your view, what in general do internet discussions say about GM foods?		2.79	0.92		
Food additives	In your view, how do netizens feel about food additives?		2.54	0.93	M = 2.63 SD = 0.81 α = 0.75	
	In your view, what in general do internet discussions say about food additives?		2.71	0.89		
Perceived present opinion						
GM foods	What do you think is the opinion of the majority of the Chinese people about GM foods/the use of food additives?	1 = Strongly unfavorable, 5 = Strongly favorable	2.64	0.90		
Food additives			2.64	0.88		
Perceived future opinion						
GM foods	What do you think the general opinions of the Chinese people about GM foods/the use of food additives will be in 10 years?	1 = Strongly unfavorable, 5 = Strongly favorable	3.11	1.23		
Food additives			2.94	1.16		
Perceived opinion of important others						
GM foods	What do you think about the opinions of the people around you (e.g., family, friends) about GM foods/the use of food additives?	1 = Strongly unfavorable, 5 = Strongly favorable	2.46	0.97		
Food additives			2.54	0.98		
Perceived opinion of experts						
GM foods	What do you think of the opinions of experts (e.g., government agencies, university research institutes, and corporate research institutes) on GM foods/the use of food additives?	1 = Strongly unfavorable, 5 = Strongly favorable	3.30	0.88		
Food additives			3.16	0.85		
Self-opinion						
GM foods	How do you feel about GM foods, in general?	1 = Strongly unfavorable, 5 = Strongly favorable	2.55	0.95		M = 2.60 SD = 0.70 α = 0.77
	I do not consider buying any products containing genetically modified components a wise decision. (reverse)	1 = Definitely will not, 5 = Definitely will	2.73	1.06		
	I think buying organic products instead of GM foods is a better option, regardless of its price. (reverse)	1 = Prefer not to by GM foods, 5 = Prefer to buy GM foods	2.11	0.94		
	I think GM foods are ethically problematic. (reverse)	1 = Problematic, 5 = Not problematic	2.56	0.91		
	I agree with using genetic recombination technology in food manufacturing.	1 = Strongly disagree, 5 = Strongly agree	3.04	1.01		
Food additives	How do you feel about the use of food additives in general? I’m positive about the use of food additives. I agree with using proper amount of additives in food. I think using food additives to preserve food from decay is essential. I think buying foods with less additives is better.	1 = Strongly disagree, 5 = Strongly agree	2.67 2.71 3.35 3.40 3.76	0.87 0.99 0.98 0.95 0.85		M = 3.18 SD = 0.68 α = 0.79
Self-relevance						
GM foods	I see a close connection between me and GM foods issue. GM foods issue has serious consequences for my life and someone I care.	1 = Strongly disagree, 5 = Strongly agree	4.01 4.14	0.85 0.77		M = 4.08 SD = 0.71 α = 0.71
Food additives	I see a close connection between me and food additives issue. Food additives issue has serious consequences for my life and someone I care.		4.09 4.21	0.80 0.74		M = 4.15 SD = 0.66 α = 0.65
Self-influence	I can lead others to my point of view if I want. I tend to change the minds of others without being too hard. I enjoy discussing with people who have different views. I usually win when discussing with people who have a different opinion. I think I am well-connected with other people.	1 = Strongly disagree, 5 = Strongly agree	3.67 2.96 3.89 3.31 3.70	0.77 0.93 0.78 0.82 0.86		M = 2.03 SD = 0.72 α = 0.73
Willingness to speak out						
GM foods	If I discover a controversial discussion on GM foods on the social media, I will be willing to compose original posts and give my opinion. If I discover a controversial discussion on GM foods on the social media, I am willing to forward posts of GM foods. If I discover a controversial discussion on GM foods on the social media, I am willing to comment on posts of GM foods.	1 = No, 2 = Unsure, 3 = Yes	2.06 1.85 1.79	0.88 0.89 0.89		M = 2.26 SD = 0.57 α = 0.61
Food additives	If I discover a controversial discussion on the use of food additives on the social media, I will be willing to compose original posts and give my opinion. If I discover a controversial discussion on the use of food additives on the social media, I am willing to forward posts of food additives. If I discover a controversial discussion on the use of food additives on the social media, I am willing to comment on posts of food additives.		2.04 1.80 1.75	0.87 0.89 0.89		M = 2.28 SD = 0.56α = 0.60

Note: GM food = Genetically modified food.

**Table 3:** Factors influencing opinion perceptions.

	Present Opinion		Future Opinion		Important Others’ Opinion		Experts’ Opinion	
	GM Food	Additive	GM Food	Additive	GM Food	Additive	GM Food	Additive
Netizen opinion	0.51 ***	0.57 ***	0.09 **	0.15 ***	0.47 ***	0.55 ***	0.19 ***	0.23 ***
Self-opinion	0.19 ***	0.19 ***	0.5 ***	0.43 ***	0.33 ***	0.23 ***	0.28 ***	0.32 ***
Self-relevance	−0.07 **	−0.06 **	−0.03	−0.03	−0.08 **	−0.06 **	0.08 **	0.05
Gender (ref. = male)	0.01	0.01	0.03	0.01	−0.05 *	−0.02	−0.02	−0.03
Age	−0.08 **	0.02	−0.14 ***	−0.10 ***	−0.01	0.02	−0.10 ***	−0.04
Education	0.04	−0.03	−0.03	0.01	0.04	−0.01	−0.01	0.01
Income	0.02	0.01	0.03	−0.05	0.02	−0.02	−0.05	−0.05
Total Adj. R^2^ (%)	41.0	45.2	34.6	27.3	47.6	46.8	16.1	21.4

Note: Netizen opinion, present opinion, future opinion, important others’ opinion, and experts’ opinion all refer to perceptions of opinions. Standardized regression coefficients are reported. GM food = Genetically modified food. * *p* < 0.05. ** *p* < 0.01. *** *p* < 0.001.

**Table 4:** Factors influencing willingness to speak out about food safety issues on social media.

DV = Willingness to Speak Out		GM Food	Additive
Independent variables	Opinion incongruence	−0.07 *	−0.05
	Self-relevance	0.15 ***	−0.10 **
	Self-influence	0.21 ***	0.17 ***
Control variables	Gender (ref. = male)	0.03	−0.01
	Age	−0.04	0.03
	Education	0.02	0.01
	Income	0.06	−0.05
	Total Adj. R^2^ (%)	8.9	5.1

Note: Standardized regression coefficients are reported. GM food = Genetically modified food. * *p* < 0.05. ** *p* < 0.01. *** *p* < 0.001.

## Data Availability

The data presented in this study are available on request from the corresponding author. The data are not publicly available due to privacy reasons.
